# A Zwitterionic Copolymer as Rheology Modifier and Fluid Loss Agents for Water-Based Drilling Fluids

**DOI:** 10.3390/polym13183120

**Published:** 2021-09-16

**Authors:** Xianfeng Tan, Longchen Duan, Weichao Han, Ying Li, Mingyi Guo

**Affiliations:** 1 Faculty of Engineering, China University of Geosciences, Wuhan 430074, China; geotan1977@126.com; 2 Lunan Geo-Engineering Exploration Institute Shandong Province, Yanzhou 272100, China; 3 Department of Construction Engineering, Jilin University, Changchun 130021, China; hanwc19941205@163.com (W.H.); yingl20@mails.jlu.edu.cn (Y.L.); 4 Shanxi Survey Design Research Institute Co., Ltd., Taiyuan 030000, China

**Keywords:** zwitterionic copolymer, rheology modifier, fluid loss agents, drilling fluid

## Abstract

To overcome the negative impact on the rheological and filtration loss properties of drilling fluids caused by elevated temperature and salts contamination, which are common in ultradeep or geothermal drilling operations, it is imperative to develop highly efficient additives used in the water-based drilling fluid. In this study, a zwitterionic copolymer P (AM/DMC/AMPS/DMAM, ADAD) was synthesized by using acrylamide (AM), cationic monomer methacrylatoethyl trimethyl ammonium chloride (DMC), anionic monomer 2-acrylamide-2-methyl propane sulfonic acid (AMPS), and *N*,*N*-dimethylacrylamide (DMAM) through free radical copolymerization. The copolymer was characterized by ^1^H Nuclear Magnetic Resonance (NMR), Fourier transform infrared spectroscopy (FTIR), elemental analysis, thermogravimetric analysis (TGA), and zeta potential. The rheological behavior, filtration properties, and the performance exposure to salt or calcium contamination in water-based drilling fluid were investigated. The bentonite/polymer suspension showed improved rheological and filtration properties even after aging at 160 °C or a high concentration of salt and calcium. The filtration loss can be greatly reduced by more than 50% (from 18 mL to 7 mL) by the inclusion of 2.0 wt% copolymer, while a slight increase in the filtrate loss was observed even when exposed to electrolyte contamination. Particle size distribution and zeta potential further validate the idea that zwitterionic copolymer can greatly improve the stability of base fluid suspension through positive group enhanced anchoring on the clay surface and repulsion force between negative particles. Moreover, this study can be directed towards the design and application of zwitterionic copolymer in a water-based drilling fluid.

## 1. Introduction

The utilization of drilling fluids, a vital part and the lifeblood of a drilling operation, has never been more important than it is today in light of the deep wells drilling for exploration of fossil fuels and geothermal energy [1,2]. Drilling fluid was applied to cool the drilling bit, transport the formation cuttings from the downhole to the surface, balance the formation pressure, inhibit shale dispersion, and prevent the invasion of fluid into formations in the drilling operations [3]. Up to now, water-based drilling fluids have mostly been recommended due to their environmentally-friendly, easy preparation, cost-effective, and excellent rheological properties compared to other kinds of drilling fluids.

Currently, bentonite (Bent) has been the primary additive adopted to form stable suspension in water-based drilling fluids [4,5]. While exposed to high temperature or high concentration electrolyte, the hydration, swelling, and dispersibility of Bent particles were greatly deteriorated. The failure of rheological property and increase of filtration loss caused by aggregation (flocculation) or thermal induce swelling of Bent particles usually lead to wellbore instability and borehole accidents, especially in a deep well or geothermal drilling operation. Besides the high temperature induced deterioration of rheological properties of clay dispersion, electrolyte contamination is another major issue related to the property deterioration for water-based drilling fluids [6].

To maintain the desired properties of water-based drilling fluid, mainly regarding rheological performance and minimized water loss into the permeable formation, much effort has been devoted over the past two decades to synthesizing various organic additives such as synthetic copolymers, modified starch [7,8,9,10], polysaccharides, and cellulose [11,12,13,14,15]. Among these additives, synthetic copolymers are widely used as shale inhibitors [16,17,18], viscosifiers, and filtration loss control agents via improving the incorporation between Bent particles and copolymer molecules [19]. The interactions between clay and copolymer significantly affect the properties of Bent/polymer suspensions. The adsorption of copolymer on clay platelets prevents the clay platelets from forming aggregates and the adsorption of copolymer on boreholes via forming a thin layer, and thus prevent the invasion of water into the formations [20].

Generally, the adsorption of polymer on the clay particles surface depends on the polymer molecule structure, the functional group of monomers in the backbone, and the charge distribution in monomer groups [21,22,23]. Moreover, most copolymers used in water-based drilling fluid are sensitive to high temperatures and high concentrations of electrolytes in solutions, which could severely affect the viscosity and filtration loss properties. To overcome these problems, copolymers with thermal and electrolytes resistant monomers or inflexibility groups, such as 2-acrylamide-2-methylpropanesulfonic acid (AMPS) and other monomers with the SO_3_^−^ group, have received considerable attention [24]. Recently, numerous copolymers that contain AMPS monomer have been prepared and applied as a viscosity modifier and fluid loss control additives in water-based drilling fluids. Furthermore, to strengthen the anchor force of the copolymer on the negative surface of Bent platelets, positively charged monomers could be introduced into the copolymer chains for a better thermal and electrolytes resistance ability [25]. But so far, whereas the zwitterionic copolymer containing both positive and negative groups in the chain has been reported, copolymers used for drilling in high temperature and high concentration of electrolytes formation have seldom been explored. Terpolymer prepared with AM, DMC, and AMPS has been explored as an encapsulator in deepwater drilling fluid; however, temperature and salt resistance properties have not been discussed [26,27,28]. Thus far, to obtain a water-based drilling fluid with enhanced rheological and filtration properties which can withstand high-temperature and high-salinity, polymeric additives with versatile functional groups are still desperately needed.

In this present study, a zwitterionic copolymer was synthesized by using AM, cationic monomer methacrylatoethyl trimethyl ammonium chloride (DMC), anionic monomer AMPS, and *N*,*N*-dimethylacrylamide (DMAM) through free radical copolymerization. In this copolymer, AM and DMC units act as adsorption groups and AMPS units act as thermal and electrolyte resistant hydration groups. Furthermore, to enhance the hydration property and polymerization of positively charged monomer and negatively charged monomer, the mole fraction of DMC and AMPS in the copolymer need to be optimized. The copolymer was characterized by Fourier transform infrared spectroscopy (FTIR), elemental analysis, thermogravimetric analysis (TGA), and zeta potential. The rheological behavior, filtration properties, and the performance exposure to salt or calcium contamination in water-based drilling fluid were investigated. The Bent/polymer suspension showed improved rheological properties and filtration properties even after aging at 160 °C or a high concentration of electrolytes. The mechanism of salt and temperature resistance properties was analyzed and discussed with the aid of particle size analysis and SEM.

## 2. Experimental

### 2.1. Materials and Methods

The Acrylamide (AM) (CP) was purchased from Shanghai Sanpu Chemical Compan (Shanghai, China). 2-acrylamide-2-methylpropanesulfonic acid (AMPS) (AR, 98%), methacrylatoethyl trimethyl ammonium chloride (DMC) (AR, 75% in H_2_O), and *N*,*N*-dimethylacrylamide (DMAM) (AR) were purchased from Aladdin Reagent Company (Shanghai, China). Potassium persulfate was purchased from Sinopharm Chemical Reagent Co., Ltd. (Shanghai, China). Sodium bentonite, following the American Petroleum Institute (API) standard, was obtained from Rongchang Mining Industry Bentonite Co., Ltd. (Jianping, China). The commercial sodium bentonite consists of 84.9 wt% montmorillonite ((Na,Ca)_0.33_(Al,Mg)_2_[Si_4_O_10_](OH)_2_·nH_2_O), 5.2 wt% quartz, 2.9 wt% Orthoclase, 3.0 wt% plagioclase, and 4.0 wt% calcite.

^1^H NMR spectra were recorded on a 300 MHz Nuclear Magnetic Resonance spectrometer, (Varian, CA, USA). Fourier transform infrared (FTIR) spectra were collected on a Nicolet is 10 spectrometer (Thermo Fisher Scientific Corporation, MA, USA) in the range of 400–4000 cm^−1^, the spectrum obtained with 16 scans at resolution of 4 cm^−1^. Thermogravimetry (TG) was performed on a Netzsch STA 449C thermal analyzer system (NETZSCH-Gerätebau GmbH, Serb, Germany) from room temperature to 700 °C in N_2_, at a heating rate of 10 °C/min. Elemental analysis was carried out on Perkin-Elmer ICP-OES Optima 3300DV (Perkinelmer, Massachusetts, USA). The Zeta potential and particle size distribution were performed at Zetasizer Nano ZS 90 (Malvern Panalytical, Malvin, UK). To ensure accuracy, the test was carried out in triplicate for each sample. The scanning electron microscope (SEM) was performed with an S-4800 field emission scanning electron microscope (Hitachi Limited, Tokyo, Japan).

### 2.2. Synthesis of Copolymer P(AM-DMC-AMPS-DMAM)

The copolymer P(AM/DMC/AMPS/DMAM) (ADAD) was prepared by using acrylamide (AM), cationic monomer methacrylatoethyl trimethyl ammonium chloride (DMC), anionic monomer 2-acrylamide-2-methylpropanesulfonic acid (AMPS), and *N*,*N*-dimethylacrylamide (DMAM) through free radical copolymerization. The homogeneous copolymerization was carried out in deionized water. In a typical procedure, a total of 20 g of monomers with a certain mole ratio of AM, AMPS, DMC, and DMAM was dissolved in 80 g distilled water in a three-neck flask at room temperature. After the pH was adjusted to 7–8 with NaOH solution, the mixture was stirred under an inert N_2_ atmosphere for 30 min. Then, the potassium persulfate was dropped into the above mixed solution to trigger the copolymerization, wherein the mass of potassium persulfate is 0.1 wt% of the monomers. Then, the temperature was maintained at 75 °C for 4 h to obtain the product. During the reaction, the temperature of the system was even increased to 95 °C. Then, the product was obtained by being extracted with acetone. The resulting sample was dried at 80 °C in an oven and the dried product was ground to powder. The optimized monomer ratio was determined according to the effects of monomer ratio on rheology and filtration loss performance. Then, the product of the optimal formulation was noted as PADAD; the optimal monomer mole ratio was 4:1:4:1 of AM, DMC, AMPS, and DMAM.

### 2.3. Performance Evaluation of PADAD

Base fluid (BF) was prepared by dispersing 20 g bentonite in 500 mL deionized water to form 4 wt% suspension and stirring 2 h, followed by static hydration for 24 h. A certain amount of copolymer was dissolved in the Bent suspension with 11,000 rpm stirring for 20 min. For salt contamination, specified NaCl or CaCl_2_ was added into the above suspension with 11,000 rpm stirring for 20 min, followed by static standing for 12 h for further trials. The aging procedure of mud was carried out in a rolling oven at 160 °C for 16 h. The rheological properties of Bent mud with and without copolymer were measured with a six-speed rotating viscometer Fann 35 type ZNN-D6 viscometer (Haitongda Company, Qingdao, China). The rheological performance such as shear stress (τ) was measured as a function of the shear rate (γ) over a shear rate range of 5.11 s^−1^ to 1024 s^−1^ at 25 °C in deionized water and saltwater.

The rheological parameters, such as apparent viscosity (AV), plastic viscosity (PV), and yield point (YP) were measured according to the API recommended practice by using a six-speed rotational-type rheometer (ZNN-D6). The drilling fluid was agitated at various shear rates starting from 3 to 600 rpm, and the shear stress was measured at each shear rate. The relationship between the shear rate and rotating speed can be calculated from the following equations:

(1) $$1\;\mathrm{rpm}=1.703{\;s}^{-1}.$$

The shear stress (τ) can also be calculated from the dial reading (*φ_N_*, dial reading at the rotating speed of N rpm) of the viscometer by the following equations:

(2) $$\tau =0.511\varphi_{N}.$$

The rheological parameters were calculated as the API recommended procedures for the Bingham plastic model by using 300 and 600 rpm dial readings:

(3) AV=φ6002 (mPa·s)

(4) $$\mathrm{PV}=\varphi_{600}-\varphi_{300}\;\left(\mathrm{mPa}\cdot s\right)$$

(5) $$\mathrm{YP}=0.511\times \left(2\times \varphi_{300}-\varphi_{600}\right)\;\left(\mathrm{Pa}\right)$$

The plugging performance of the copolymer was tested through the filtration of water-based mud (4 wt%). The procedure followed the API standard, which was performed at room temperature under a pressure level of 0.69 MPa. The fluid loss of water-based fluids was carried out by dissolving various concentrations of the copolymer.

## 3. Results and Discussion

### 3.1. Characterization of the Copolymer

**Figure 1:**
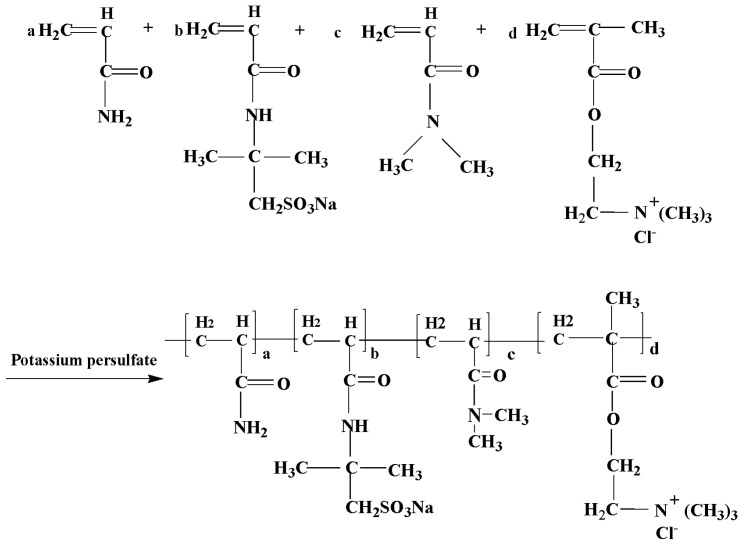
Reaction schema of the copolymer PADAD.

**Figure 2:**
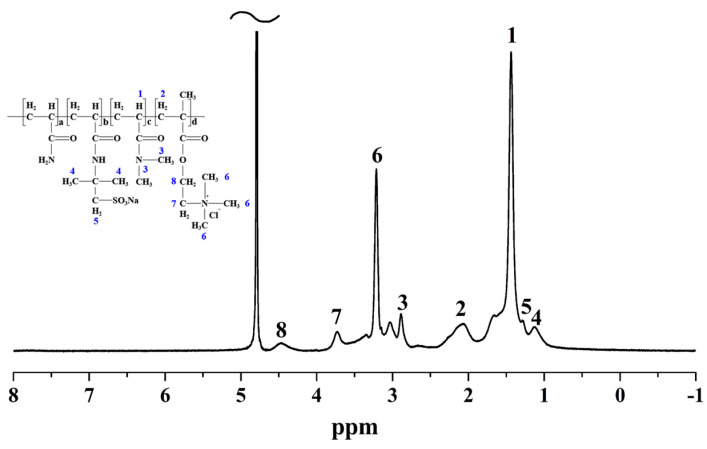
^1^H NMR spectrum of PADAD.

The proposed chemical structure of PADAD synthesized through free radical copolymerization is shown in Figure 1. To clarify the structure of the copolymer, ^1^H NMR spectrum, FTIR, and ultimate analysis were performed. The 1H spectra of PADAD were shown in Figure 2. Several characteristic peaks were seen in the spectrum of PADAD because of different monomers. The −CH_2_−CH−CO− and −CH−CO− in Acrylamide, AMPS, or DMC and DMAM were at the regions of 1.22−1.85 and 1.85−2.45 ppm, respectively. The CH_3_− in *N*,*N*-dimethylacrylamide appeared at 2.88 ppm. The −CH_2_−N^+^ and CH_3_−N^+^ in DMC could be observed at the region of 3.21and 3.35 ppm, and the peak at 3.04 ppm and 1.12 ppm was assigned to −CH_2_−SO_3_^−^ and NH-(CH_3_)_2_− in AMPS. Thus, the ^1^H NMR spectra validated the idea that the P(AM/DMC/AMPS/DMAM) copolymer was obtained by homogeneous polymerization.

**Figure 3:**
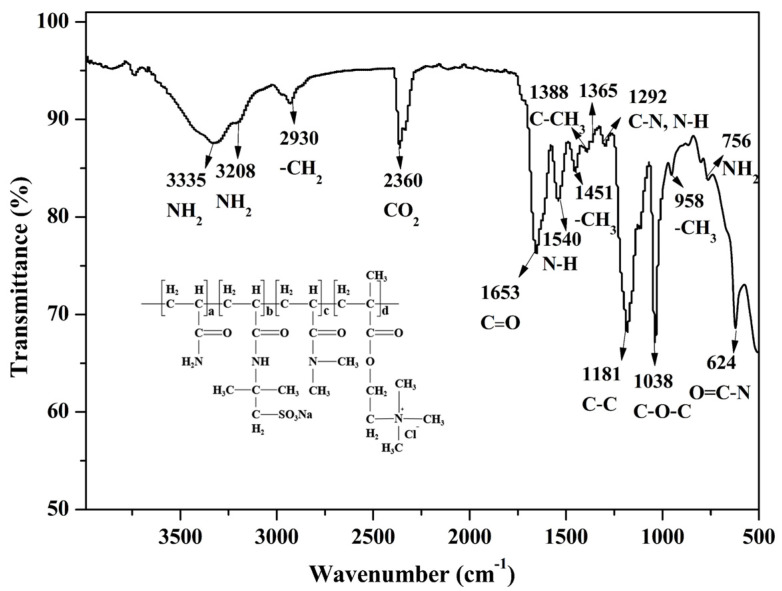
FTIR spectrum of copolymer PADAD.

The FTIR spectrum of PADAD is depicted in Figure 3. The spectrum displayed distinctive absorption bands for amidogen (NH_2_), methyl (CH_3_), methylene (CH_2_), and carbonyl(C=O) as the main functional groups in PADAD. The vibration bands at 3335 and 3208 cm^−1^ were observed due to the asymmetric and symmetric stretching vibration of NH_2_, respectively. Also, the band at 756 cm^−1^ was due to the twisting vibration of NH_2_. The absorption band at 2930 cm^−1^ was due to the asymmetric stretching vibration of CH_2_, while the absorption bands at 1451, 1388, and 1365 cm^−1^ were due to the deformation vibration of CH_3_, while the band at 958 cm^−1^ was due to the CH_3_ rocking vibration mode of the quaternary ammonium ion –N^+^(CH_3_)_3_. Moreover, the absorption band at 1653 cm^−1^ showed the existence of C=O functionality in PADAD. The band at 1540 cm^−1^ was associated with the deformation vibration of N-H of secondary amide and the C–N stretching band, and the band at 1292 cm^−1^ was a coupling band of the N–H in-plane bending vibration and C–N stretching vibration. The stretching vibrating band at 1181 cm^−1^ could be assigned to the C-C skeleton of PADAD, and the intense peak at 1038 cm^−1^ was related to the symmetric stretching vibration of C–O–C. Moreover, the 624 cm^−1^ was associated with the O=C–N band in PADAD.

The ultimate analysis (C, H, N, S) used to identify the copolymer composition is present in Table 1. The content of carbon is 37.38 wt% in the copolymer, the hydrogen, nitrogen, and sulfur are about 6.60, 8.07, and 8.28 wt%, respectively. According to the optimal monomer molar ratio of AM, DMC, AMPS, and DMAM (4:1:4:1), the mass ratio of C to N was calculated to be 4.38: 1. The ultimate analysis result was 4.63:1, which is consistent with the theoretical results.

**Figure 4:**
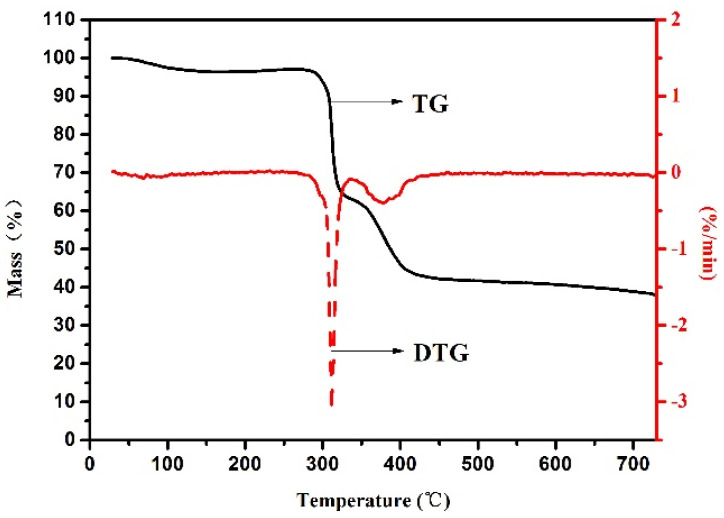
TG analysis of copolymer under nitrogen atmosphere.

To demonstrate the thermal stability of PADAD copolymer, TG analysis of PADAD was performed in N_2_ atmosphere and the result is shown in Figure 4. The weight loss of PADAD can be divided into three stages: about 4.51% of weight loss from 40 to 150 °C corresponded to free water in the copolymer, while the weight loss was about 35.2% between 290 to 330 °C which was related to the decomposition of quaternary ammonium group and amide group; it then decreased about 20.8% above 330 °C, which could be assigned to the decomposition of sulfonic groups and C–C bonds in the main polymer chains. Therefore, the superior thermal stability of PADAD makes it a promising candidate for application in the high-temperature drilling fluid.

### 3.2. Rheological Performance of Copolymer-Loaded Drilling Fluid Formulations

To investigate the rheological properties and flow behavior of Bent/polymer suspension, a widely used Herschel–Bulkley (HB) model was applied [3,29]

(6) $$\tau =\tau_{0}+K\gamma^{n}$$

where the τ, τ_0_ (Pa), *K* (Pa·s^n^), and n are shear stress, yield strength, flow consistency index, and flow behavior index, respectively. The statistical indicators coefficient of determination R^2^ was determined and listed in Table 2. It is well documented in the literature that most of the water-bentonite suspensions fitted very well with the HB model.

**Figure 5:**
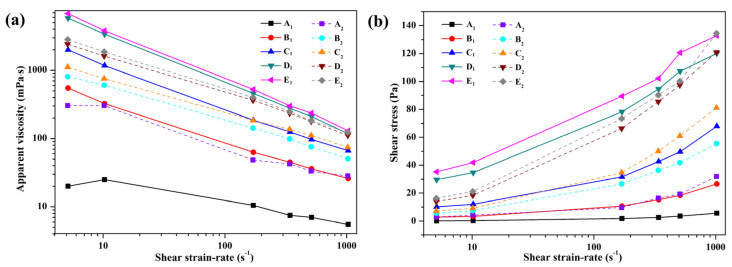
Apparent viscosity as a function of shear rate (**a**), and flow curves (**b**) of base fluid containing different concentrations of PADAD before (A_1_-E_1_) and after aging (A_2_-E_2_).

**Figure 6:**
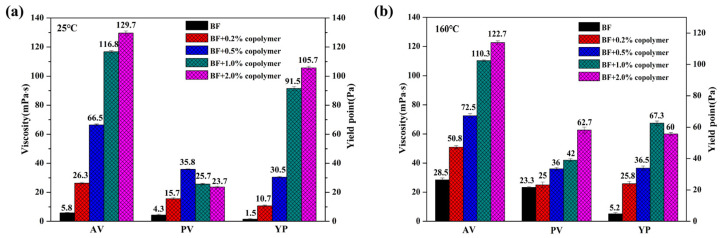
Rheological properties of drilling fluids with different concentrations of copolymer before (**a**) and after hot rolling (**b**).

Rheological properties of BF and BF/copolymer suspensions in different concentrations of copolymer before and after hot rolling are shown in Figure 5 and Figure 6. All the copolymer solutions before and after aging tests showed, non-Newtonian behavior as the increase in the shear rate causes a decrease in the viscosity of the copolymer solutions. The consistency index *K* of a drilling fluid, which greatly affected the cuttings’ transport velocity in the wellbore, is a rheological property related to the cohesion of the particles in the suspension and flow resistance. As shown in Table 2, along with the copolymer concentration increasing, the *K* increased before and after aging at high temperature, but little change was observed when the concentration exceeded 1.0 wt% either before or after aging. It was further verified that high thermal stability and strong adsorption of copolymer occur on Bent platelets. The consistency index *K* of BF and BF/copolymer before aging showed results of 0.309, 1.056, 4.630, and 5.136, respectively. After aging at 160 °C, the consistency index changed to 1.273, 1.411, 3.712, and 3.905, respectively.

Enhanced shear-thinning ability is important for cutting transport and hole cleaning. As shown in Table 2, the τ_0_ of the BF with different concentrations of copolymer before thermal aging is 2.81, 10.22, 29.63, and 35.25, respectively. Meanwhile, the τ_0_ of BF/copolymer was found to be 4.34, 5.87, 12.26, and 14.30 after aging at 160 °C. Compared to the consistency index, although it obviously deceased as the increase of copolymer concentration occurred, little change of the flow behavior index *n* after hot-rolling indicated its excellent thermal stability.

It was noted that the apparent viscosity of BF/copolymer dispersions shows minuscule variation before and after hot rolling (Figure 5), particularly as the PADAD concentration is high than 0.5 wt%, which indicates the presence of AMPS monomer in quadripolymer inducing high stability towards the high temperature of BF/copolymer. As shown in Figure 6, the viscosity of the BF/copolymer increased with the increase of copolymer concentration. The AV of 4 wt% sodium bentonite suspension is 6 mPa·s at 25 °C, and it increased to 120 mPa·s with 1.0 wt% PADAD; while the polymer concentration was higher than 1.0 wt%, the viscosity of the BF/copolymer did not change significantly. The increasing trends of PV of the BF/PADAD after aging indicated that aggregation of Bent platelets might have occurred due to thermal induce dehydration. Additionally, YP, which depends on electrochemical charges in the suspensions, exhibited declining trends after hot rolling. This implied that high temperature might weaken the charge interaction between clay and PADAD, and consequently reduce the yield point.

Given the previous study indicating that the interaction between the clay and copolymers depends on a copolymer structure, the chemical composition, and charge distribution in monomer groups, it was inferred that positively charged DMC monomer enhanced the adsorption on clay via electrostatic attraction forces among negatively charged Bent platelets and copolymer chain, resulting in a strengthened network structure between clay particles and copolymers.

**Figure 7:**
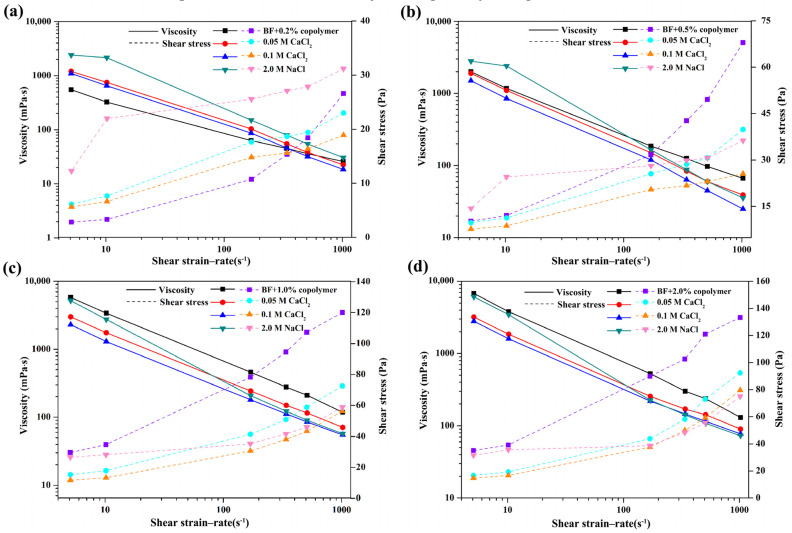
Apparent viscosity and shear stress as a function of the shear rate of BF and BF/ PADAD (**a**) 0.2 wt%, (**b**) 0.5 wt%, (**c**) 1.0 wt%, (**d**) 2.0 wt% with different concentrations of electrolytes ((NaCl or CaCl_2_) at 25 °C.

To inspect the salt contamination resistant property of the drilling fluid, the effect on viscosity of electrolyte in copolymer solutions was conducted. As shown in Figure 7, the monovalent and divalent cations, introduced by adding NaCl or CaCl_2_ into the solution, led to viscosity decrease. Generally, the electrical double layer of bentonite was affected by metal cations adsorption on the surface, resulting in a major change in viscosity due to the bentonite platelet coagulation. However, due to the adsorption of copolymer on the clay surface, the clay platelets aggregation could be greatly reduced. The AV of BF/0.2 wt% PADAD with and without 2.0 mol/L NaCl are 30 mPa·s and 25.5 mPa·s, respectively. In the concentration of 2.0 mol/L NaCl solution, the AV of BF/PADAD suspension increases from 30 mPa·s to 73 mPa·s, with the PADAD concentration between 0.2 wt% to 2.0 wt%. Similarly, when exposed to 0.05 mol/L and 0.1 mol/L CaCl_2_, the AV of BF/PADAD decreases sharply compared to blank suspension, although it still gives a high viscosity of 77.5 mPa·s with 2.0 wt% PADAD in 0.1 mol/L CaCl_2_. The results reveal that the viscosity of BF/PADAD significantly declined following the invasion of electrolyte, then stayed at an acceptable value. The curves showed similar trends in the case of NaCl and CaCl_2_ solutions. The results indicated that the salt ions had a distinct effect on the conformation of PADAD through interaction among Na^+^, Ca^2+^, and anion groups in the molecular chain. The remaining viscosity could be attributed to the electrostatic attraction between PADAD and clay enhanced by positive moieties, while the repulsive interactions among clay platelets were enhanced by the negatively charge on the surface.

**Figure 8:**
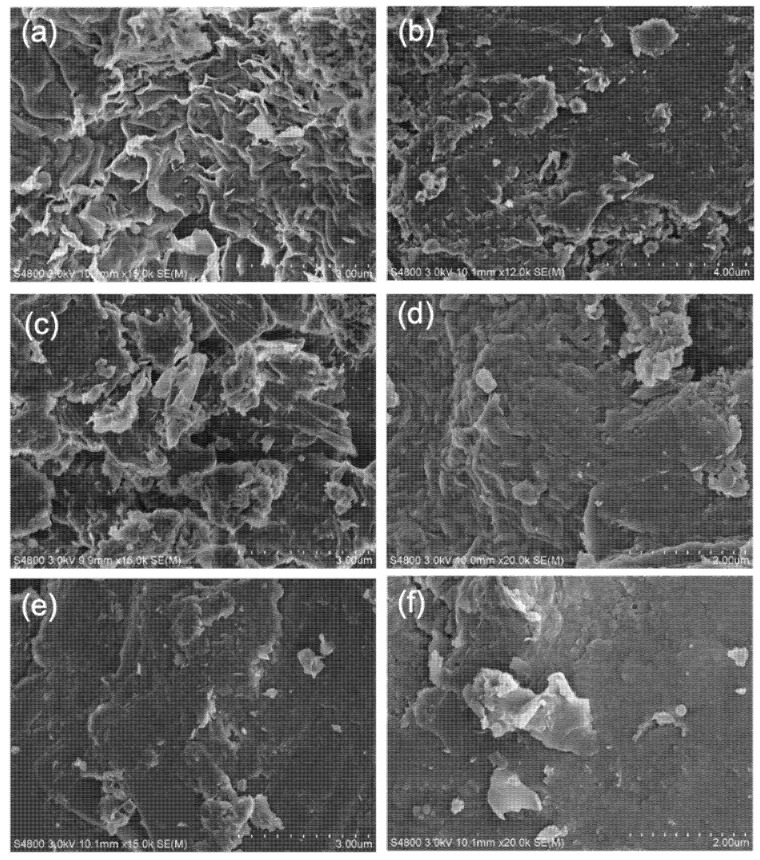
SEM images of bentonite (**a**), bentonite suspended in various concentrations of PADAD (**b**) 0.2%, (**c**) 0.5% (**d**) 1.0%, (**e**,**f**) 2.0%.

Furthermore, SEM was performed to demonstrate the adsorption of the copolymer on the bentonite particles. As shown in Figure 8, in the absence of polymer, lamella structured bentonite platelets were observed. Meanwhile in the solution containing polymer, the adsorption of the polymer on the clay resulted in a smooth surface of the particles and the individual lamellar structure could not be observed. As the polymer concentration increased, the smoother surface confirmed that the positively charged polymer could be better adsorbed on the surface of the particles, thereby effectively adjusting the clay surface properties; as a consequence, the hydration properties after high-temperature aging were retained.

### 3.3. Filtration Characteristics of BF/Copolymer Suspension

The filtration loss volume, permeability, and thickness of filter cakes strongly influence the drilling performance. Therefore, the filtration characteristics of BF and BF/copolymer suspensions were demonstrated by using the Fann filter press apparatus at 25 °C and 100 psi pressure. Generally, the filtrate volume increases linearly with the squared filtration time, which means the linear relationship between the volume and the square root of time [30]. Hence, Darcy’s law was used to determine the flow through filter cakes:

(7) $$\frac{dV_{f}}{dt}=\frac{KA\Delta P}{\mu h_{mc}}$$

(8) $$V_{f}\;dV_{f}=\frac{KA^{2}\left(\frac{f_{sc}}{f_{sm}}-1\Delta P\right)}{\mu }\;dt$$

(9) $$V_{f}=A\sqrt{2KA\Delta P\left[\frac{f_{sc}}{f_{sm}}-1\right]}\frac{\sqrt{t}}{\sqrt{\mu }}.$$

But before the filter cake is established, the spurt-loss volume should not be neglected. When considering the spurt-loss volume, the formula can be presented by:

(10) $$V_{f}-V_{sp}=A\sqrt{2KA\Delta P\left[\frac{f_{sc}}{f_{sm}}-1\right]}\frac{\sqrt{t}}{\sqrt{\mu }}$$

(11) $$M=A\sqrt{\frac{2KA\Delta P}{\mu }\left[\frac{f_{sc}}{f_{sm}}-1\right]}$$

(12) $$V_{f}-V_{sp}-=M\sqrt{t}.$$

The spurt-loss volume can be calculated by the following formula:

(13) $$V_{sp}=2V_{7.5}-V_{30}$$

where *K* (μD) is the permeability of the filter cake, *A* is the area of filter medium, (45.6 cm^2^), *μ* is the viscosity of the filtrate (8.9 × 10^−4^ Pa·s), *h_mc_* (cm) is the thickness of the filter cake, and ∆*P* is the pressure applied on the filter cake (0.69 MPa). The *f_sm_* is the solid volume fraction in drilling fluid and *f_sc_* is the solid volume fraction in the filter cake. Additionally, *t* (min) is the time for filtration, *V_sp_, V*_7.5_, and *V*_30_ represent spurt loss, filtrate volume in 7.5 min, and represents the filtrate volume in 30 min. *M* is the linear slope denoting the rate of fluid loss.

**Figure 9:**
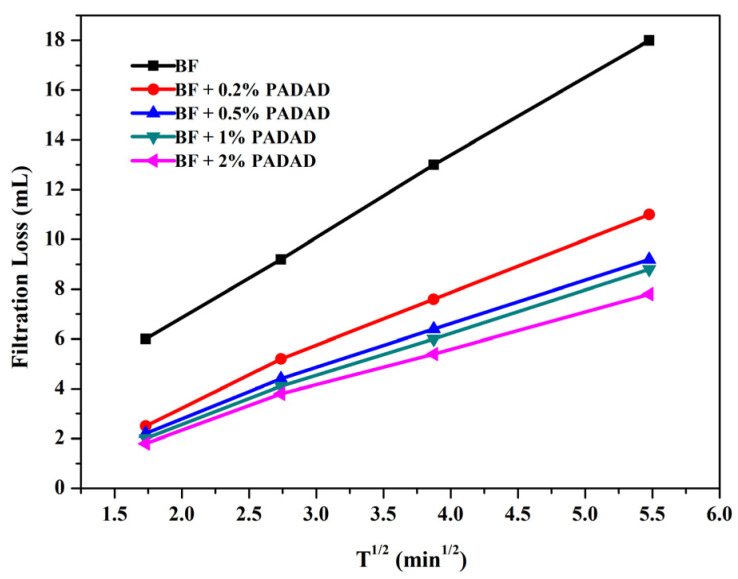
Fluid loss vs. time profiles of BF and BF/PADAD suspensions according to API procedures at 25 °C.

As shown in Figure 9, the cumulative filtrate volume as a function of the square root of time for both BF and BF/copolymer suspensions show different concentrations of the copolymer. BF suspension showed a maximum fluid loss (18 mL) compared to the other BF/copolymer suspensions which is under the acceptable range (<15 mL) according to the API standard. The incorporation of copolymers in BF suspension visibly reduced the fluid loss of all BF/copolymer suspensions. As a list in Table 3, the fluid loss of BF/copolymer suspensions with copolymer concentrations of 0.2, 0.5, 1.0, and 2 wt% was 11.0 mL, 9.2 mL, 8.8 mL, and 7.8 mL, respectively. Compared to the BF suspension, a reduction was up to 56.7% for BF/copolymer suspension with 2.0 wt% copolymer.

When simplifying the relationship between the filtration rate and time according to Formula (10), *M* represents the slope of the linear, which reflects the rate of fluid loss. It can be seen that the rate of fluid loss during the filtration test was high at the beginning for all the suspensions due to the absence of thin filter cake at the start of filtration. Then the rate of fluid loss decreased due to the formation of filter cake. The slope of the linear M was recorded as 3.176, 1.935, 1.643, 1.570, and 1.460 with the copolymer content as 0, 0.2, 0.5, 1.0, and 2 wt%, respectively. The mitigation of spurt loss (*V_sp_*) could also be observed by the addition of copolymer, which indicated that the enhanced corporation of polymers with Bent palates formed a dense filter cake. The *V_sp_* was 0.6, 0.4, 0.2, 0.2, and 0.1 mL when the copolymer content was 0, 0.2, 0.5, 1.0, and 2 wt%, respectively. Moreover, the increase of *K* and decrease of *n* indicated that the filtration loss was significantly improved, which can effectively reduce mud invasion into the formation and thus strengthen wellbore stability.

**Figure 10:**
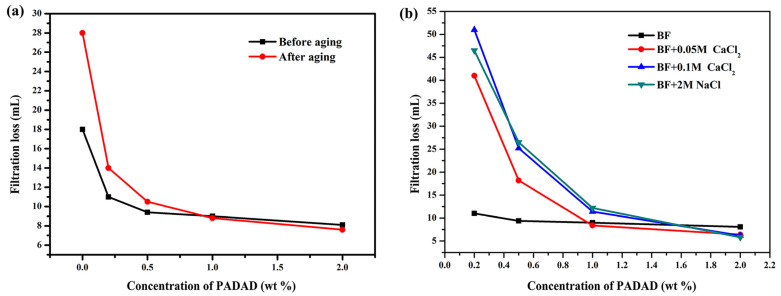
(**a**) The filtration loss (FL_API_) of BF with different PADAD concentrations before and after aging at 160 °C for 16 h. (**b**) The filtration loss (FL_API_) of BF with different PADAD concentrations in the presence of NaCl or CaCl_2_.

To assess the high-temperature filtration loss of BF/copolymer suspension, the tests were conducted before and after aging at 160 °C. As presented in Figure 10a, a great increase of filtration loss (from 18 mL to 28 mL) and thick filter cakes of BF could be observed after hot rolling, which could be attributed to the flocculation of Bent platelets at higher temperature conditions. But the BF/copolymer suspensions showed almost the same filtration loss as before aging. In particular, the filtration loss of BF/copolymer suspensions with 1.0 wt% and 2.0 wt% copolymer could be reduced to less than 10 mL after hot aging. Furthermore, only a slight increase in the filtrate loss was observed of BF/copolymer suspensions when exposed to electrolyte contamination. Although high filtration loss (more than 15 mL) could be seen in the concentrations of 0.2 wt% and 0.5 wt%, as the copolymer concentration was 0.2 wt%, the FL_API_ of BF/copolymer was 41, 51, and 45 mL in 0.05 and 0.1 M calcium chloride and 2 M sodium chloride, respectively (Figure 10b). The filtration loss was decreased lower than 15 mL even in 0.1 mol/L CaCl_2_ or 2 mol/L NaCl solution when the polymer concentration was higher than 1.0 wt%. As the polymer concentration was 1.0 wt%, the FL_API_ was 8.4, 11.2, and 12.2 mL in 0.05 and 0.1M calcium chloride and 2M sodium chloride, respectively. The results further implied that, though high temperatures and electrolytes may cause particles to flocculation by reducing the clay hydration or hindering electrostatic repulsion among the platelets, the zwitterionic copolymer can effectively protect the hydration of particles by adsorbing tightly on the clay surface via electrostatic forces between positive charges on functional groups of the copolymer and negatively charged bentonite platelets, resulting in appropriate size distribution and a strong negative charged surface of clay particle forming a compacted mud cake.

### 3.4. Mechanism Analysis of High-Temperature-Resistant Filtrate Reducer

**Figure 11:**
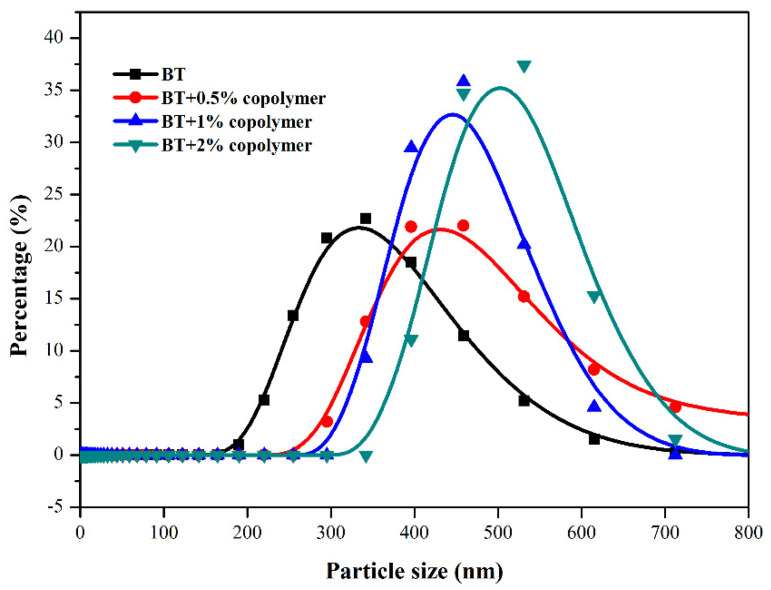
Particle size distribution of clay suspension with different concentrations of the copolymer.

The copolymers displayed remarkable filtration reduction results in water-based drilling fluids, either before or after high temperature rolling. To clarify the relationship between molecular structure and performance, the zeta potential and size distribution analysis of the Bent/polymer suspension were discussed. Firstly, the copolymer dissolved in aqueous solution can effectively improve liquid phase viscosity by forming a spatial network structure, followed by being combined with bentonite particles in the filtration cake, and thus restraining water molecules to some extent to decrease the filtration loss. Secondly, the cationic quaternary ammonium group on the zwitterionic copolymers branches facilitates the strong adsorption on the negatively charged bentonite surface by electrostatic interaction. To ensure low filtration loss either after hot aging or exposure in high concentration electrolyte contamination, appropriate particle size distribution is necessary to form a compacted filter cake. The particle size distribution of clay suspension with different concentrations of the copolymer was presented in Figure 11. The mean particle size of BF suspension is about 320 nm while the mean diameter of clay platelets is increased with the increasing of copolymer concentration, implying the effective adsorption of polymer on the clay surface. The diameter of Bent with 2.0 wt% PADAD ranges from 300 nm to 700 nm were observed. Moreover, a narrow particle size distribution could be seen for all copolymer added suspensions.

To understand the variation of surface charge of Bent/polymer platelets, which links to diffuse double layer of the clay hydration, the zeta potential was evaluated at pH 7.0. As shown in Table 4, the average zeta potential of the BF was recorded as −31.9 mV, and the zeta potential of the copolymer was −41.9 mV due to the sulfonic group in the chain. Notably, the reduction of average zeta potential was observed for all BF/copolymer suspensions compared to BF suspension, resulting in the repulsive interactions among negatively charged Bent platelets being enhanced. Thus, the aggregation of the clay platelets was prevented due to the increased repulsion force between negative clay/polymer particles. The zeta potential of the BF/copolymer in the copolymer concentrations of 0.5 wt%, 1.0 wt%, and 2.0 wt% was −40.3 mV, −37.2 mV, and −41.8 mV, respectively. As the copolymer concentration was increased, the zeta potential became increasingly negative gradually to keep the suspension stable.

Therefore, the presence of positive and negative functional groups in the zwitterionic copolymer can greatly improve the physical stability of Bent/polymer suspension through positive group enhanced anchoring on the clay surface and repulsion force between negative clay/polymer particles.

## 4. Conclusions

A zwitterionic copolymer PADAD was synthesized by using AM, DMC, AMPS, and DMAM monomers through aqueous free-radical copolymerization. The optimal reaction parameters were determined and the molecular structure and thermal stability of PADAD were confirmed by FTIR and TG analysis, indicating that the degradation temperature of the copolymer was above 290 °C. The rheological and filtration loss properties of BF/PADAD demonstrated that the copolymer has a high thermal resistance and salt tolerance. The fairly comparable apparent viscosity of the BF/PADAD as the concentration higher than 0.5 wt% before and after hot rolling further confirmed the improved rheological property by inclusion PADAD. Meanwhile, it was effective in controlling fluid loss after aging at high temperature as the concentration of PADAD was higher than 1.0 wt%. Moreover, the PADAD reduced API filtration loss by forming a tight filtrate cake. Particle size distribution and zeta potential further validated that the enhanced rheology behavior and filtration loss reduction performance could be attributed to the presence of positive and negative functional groups in the zwitterionic copolymer through positive group enhanced anchoring on the clay surface and repulsion force between negative clay/polymer particles. Therefore, this work can promote the application of zwitterionic copolymer in water-based drilling fluids. Zwitterionic copolymer prepared by monomers with multiple functional groups has great potential as the additives used to adjust the drilling fluids performance. Additionally, deep insights into the mechanism of interaction between the functional groups and clay particles should be explored in further study.

## Figures and Tables

**Table 1:** Ultimate analysis of copolymer PADAD.

Sample	C (wt%)	H (wt%)	N (wt%)	S (wt%)
1	37.18	6.607	8.08	8.006
2	37.43	6.595	8.08	8.323
3	37.55	6.620	8.05	8.521
Average	37.387	6.607	8.07	8.283
Standard Deviation	0.1887	0.0125	0.0173	0.2598

**Table 2:** Rheological parameters τ_0_, *K*, and *n* of drilling fluid contain different concentrations copolymer before and after aging treatment.

Sample	Temp (°C)	Water (mL)	Bent (g)	PADAD (g)	τ_0_ (Pa)	*K* (Pa·s^n^)	*n*	R^2^
A_1_	25	350	14	0	0.102	0.043	0.700	0.997
B_1_	25	350	14	0.7	2.810	0.309	0.627	0.994
C_1_	25	350	14	1.75	10.22	1.056	0.579	0.992
D_1_	25	350	14	3.5	29.63	4.630	0.439	0.951
E_1_	25	350	14	7.0	35.25	5.136	0.435	0.951
A_2_	160	350	14	0	1.86	0.133	0.766	0.994
B_2_	160	350	14	0.7	4.343	1.273	0.524	0.984
C_2_	160	350	14	1.75	5.876	1.411	0.565	0.987
D_2_	160	350	14	3.5	12.26	3.712	0.475	0.969
E_2_	160	350	14	7.0	14.30	3.905	0.493	0.974

**Table 3:** Change in *V*_7.5_, *V*_30_, *V_sp_*, and *M* of drilling fluid containing different concentrations of PADAD.

Sample	*V*_7.5_ (mL)	*V*_30_ (mL)	*V_sp_* (mL)	*M*
BF	9.3	18	0.6	3.176
BF+0.2 wt% PADAD	5.7	11	0.4	1.935
BF+0.5 wt% PADAD	4.7	9.2	0.2	1.643
BF+1.0 wt% PADAD	5.0	8.8	0.2	1.570
BF+2.0 wt% PADAD	3.9	7.8	0.1	1.460

**Table 4:** Zeta potential of BF or BF/copolymer suspension.

Samples	Zeta_1_ (mV)	Zeta_2_ (mV)	Zeta_3_ (mV)	Zeta_ave_ (mV)
BF	−32.5	−30.6	−32.6	−31.9
BF + 0.5 wt% PADAD	−39.5	−41.3	−40.1	−40.3
BF + 1.0 wt% PADAD	−34.0	−38.9	−38.7	−37.2
BF + 2.0 wt% PADAD	−37.2	−43.3	−44.8	−41.8
PADAD	−42.1	−38.6	−44.9	−41.9

## Data Availability

The data presented in this study are available on request from the corresponding author.
